# Supplementary material for: Genome Sequencing of Lentinula edodes Revealed a Genomic Variant Block Associated with a Thermo-Tolerant Trait in Fruit Body Formation
Source: J Fungi (Basel). 2024 Sep 2;10(9):628. doi: 10.3390/jof10090628 (PMC11432811; doi:10.3390/jof10090628)
Supplement: Supplementary file 1 [file jof-10-00628-s001.zip › Tables S1-S9.pdf]

Supplementary Table S1. Summary for PacBio whole-genome sequencing of Sanmaru and Sanjo

Sample ID	SRA accession no.	No. of PacBio subreads	Total bases (Gb) (Sequencing depth, x)	Average read length (bp)
Sanmaru 1-33	SRR27051782	789,620	11.45 (248.9)	14,508
Sanjo 502-19	SRR27051781	697,851	11.90 (258.7)	17,059

Supplementary Table S2. Summary for Illumina whole-genome sequencing of Sanmaru and Sanjo

Sample ID	SRA accession no.	No. of reads	Total bases (Gb) (Sequencing depth, x)	Read length (bp)
Sanmaru 1-33	SRR23604075	43,675,686	6.60 (143.5)	100
Sanmaru 1-42	SRR23604074	50,619,778	7.64 (166.1)	100
Sanjo 502-19	SRR23604077	43,135,530	6.51 (141.5)	100
Sanjo 502-23	SRR23604076	48,548,584	7.33 (159.3)	100
M1-15	SRR23604081	47,027,922	7.10 (154.3)	100
M1-20	SRR23604080	41,400,284	6.25 (135.9)	100
M1-21	SRR23604079	49,918,970	7.54 (163.9)	100
M1-6	SRR23604078	40,206,136	6.07 (132.0)	100

Supplementary Table S3. Summary for WG *de novo* assembly of Sanmaru and Sanjo

Sample ID	Genome	No. of contigs	Total length (bp)	N50
Sanmaru 1-33	Nuclear	12	47,117,528	4,630,509
	Mitochondria	1 (circular)	121,489	NA
Sanjo 502-19	Nuclear	11	46,496,380	5,369,569
	Mitochondria	1 (circular)	121,857	NA

NA, not applicable.

Supplementary Table S4. Anchoring contigs of Sanmaru and Sanjo to Lemap 2.0

Chromosome of Lemap 2.0	Sanmaru 1-33			Sanjo 502-19		
	Scaffold ID	Length (bp)	GC (%)	Scaffold ID	Length	GC (%)
Chr 1	chr1_1	2,468,361	45.86%	chr1	6,501,783	46.06%
	chr1_2	4,359,593	45.92%			
Chr 2	chr2_1	4,630,509	46.24%	chr2_1	74,966	46.75%
	chr2_2	2,627,387	45.91%	chr2_2	1,915,317	46.19%
				chr2_3	5,228,965	46.02%
Chr 3	chr3	4,631,886	46.10%	chr3	5,369,569	46.20%
Chr 4	chr4_1	1,096,442	46.16%	chr4	5,777,197	45.92%
	chr4_2	5,026,767	46.34%			
Chr 5	chr5	5,182,872	45.92%	chr5	4,516,734	46.20%
Chr 6	chr6	3,706,928	46.34%	chr6	3,494,503	46.07%
Chr 7	chr7	6,478,101	45.96%	chr7	6,804,373	46.12%
Chr 8	chr8	2,879,021	46.78%	chr8	2,477,139	46.63%
Chr 9	chr9	4,029,661	46.05%	chr9	4,335,834	45.99%

Supplementary Table S5. Abundance of repetitive DNAs identified in the genomes of Sanmaru and Sanjo

Repeat type	Sanmaru 1-33			Sanjo 502-19		
	No. of elements	Length (bp)	Genomic fraction (%)	No. of elements	Length (bp)	Genomic fraction (%)
Retrotransposons	8,211	10,072,595	21.38	7,880	9,173,590	19.73
LINEs	459	358,498	0.76	471	341,266	0.73
LTR elements	7,751	9,715,631	20.62	7,408	8,834,469	19.00
DNA transposons	550	192,817	0.41	789	389,934	0.84
DNA	489	174,836	0.37	599	290,065	0.62
RC	61	17,982	0.04	190	100,057	0.22
Unclassified	7,291	2,749,564	5.84	7,597	2,693,682	5.79
Small RNA	56	33,334	0.07	64	28,134	0.06
Satellites	0	0	0.00	68	31,134	0.07
Simple repeats	5,983	265,466	0.56	5,841	257,614	0.55
Low complexity	1,187	69,392	0.15	1,105	62,488	0.13
Total		13,182,680	27.98		12,338,792	26.54

Supplementary Table S6. Statistics of Carbohydrate-Active enzymes identification results for Sanmaru, Sanjo and B17 genes.

Category	Sanjo 502-19		Sanmaru 1-33		B17	
Total Genes	16,380	100.0	15,900	100.0	16,570	100.0
CAZyme	685	4.18	656	4.13	647	3.90
AA (Auxiliary Activites)	109	0.67	110	0.69	106	0.64
CBM (Carbohydrate-Binding Modules)	38	0.23	35	0.22	30	0.18
CE (Carbohydrate Esterases)	44	0.27	37	0.23	37	0.22
GH (Glycoside Hydrolases)	340	2.08	317	1.99	339	2.05
GT (Glycosyl Transferases)	142	0.87	145	0.91	123	0.74
PL (Polysaccharide Lyases)	12	0.07	12	0.08	12	0.07

Supplementary Table S7. Carbohydrate-Active enzymes of Sanmaru specific orthologous gene clusters in Sanmaru (HighTm) versus Sanjo (LowTm).

CAZyme Class	Description	Gene ID	Gene Symbol	chr	start	end	strand
GT15	Glycosyl Transferases	LESMR05715.1	MNT1	ch3	4,456,875	4,458,756	+
GT4	Glycosyl Transferases	LESMR04291.1	CBPY	ch2_2	2,606,345	2,608,546	+
GH13	Glycoside hydrolases	LESMR00872.1	AMY1	ch1_2	810,290	812,161	-
GH9	Glycoside hydrolases	LESMR04826.1	GUN4	ch3	1,738,959	1,740,133	-
GH28	Glycoside hydrolases	LESMR08978.1	RHGA	ch6	148,873	154,532	+
GH5	Glycoside hydrolases	LESMR13688.1	LESMR13688.1	ch9	3,896,192	3,897,955	-
CBM50	Carbohydrate-Binding Modules	LESMR11395.1	ENLYS	ch7	4,380,678	4,381,326	-

Supplementary Table S8. Carbohydrate-Active enzymes of Sanjo specific orthologous gene clusters in Sanmaru (HighTm) versus Sanjo (LowTm).

CAZyme Class	Description	Gene ID	Gene Symbol	chr	start	end	strand
GT15	Glycosyl Transferases	LESJ01285.1	KRE2	ch1	4,243,621	4,244,447	+
GT15	Glycosyl Transferases	LESJ03644.1	MNT2	ch2_3	3,558,749	3,560,486	+
GH10	Glycoside hydrolases	LESJ01520.1	XYNA	ch1	4,912,205	4,912,767	-
GH18	Glycoside hydrolases	LESJ05564.1	CHIA1	ch3	4,234,409	4,235,946	+
GH152	Glycoside hydrolases	LESJ06610.1	CRJ35	ch4	2,451,042	2,452,683	-
GH28	Glycoside hydrolases	LESJ06680.1	PGLRX	ch4	2,634,578	2,636,472	+
GH53	Glycoside hydrolases	LESJ07176.1	GANA	ch4	4,178,953	4,180,557	-
GH51	Glycoside hydrolases	LESJ08164.1	ABFA	ch5	1,653,298	1,656,426	+
GH16	Glycoside hydrolases	LESJ08703.1	E3GAL	ch5	3,417,316	3,420,202	+
GH5	Glycoside hydrolases	LESJ09707.1	EGLB	ch6	2,230,641	2,232,668	+
GH16	Glycoside hydrolases	LESJ10943.1	YHZ7	ch7	2,564,088	2,565,595	+
GH79	Glycoside hydrolases	LESJ08568.1	LESJ08568.1	ch5	2,985,579	2,987,131	+
GH146	Glycoside hydrolases	LESJ06262.1	TBB	ch4	1,162,891	1,164,716	-
GH3	Glycoside hydrolases	LESJ09249.1	BGLA	ch6	760,158	763,202	-
CE4	Carbohydrate Esterases	LESJ04310.1	PGDAE	ch3	450,415	451,953	-
CE4	Carbohydrate Esterases	LESJ13785.1	CDA	ch9	2,800,018	2,802,027	-
AA3	Auxiliary Activities	LESJ07473.1	ALOX1	ch4	5,198,526	5,201,496	-
AA3	Auxiliary Activities	LESJ12062.1	PDH3	ch7	6,122,383	6,124,131	+

Supplementary Table S9. Candidate SNPs and Indels associated with high-temperature tolerance.

High-temperature Sensitive	High-temperature Tolerant	Zygosity	Effect	GENE	Description	Marker
A	G	Homozygous	missense	GENE00389	hypothetical protein BOTBODRAFT_177780	RL-LE-306
G	A	Homozygous	synonymous	GENE03306	ataxin-10-like protein	RL-LE-307
T	C	Homozygous	missense	slc16a10	Monocarboxylate transporter 10	RL-LE-310
C	T	Homozygous	missense			RL-LE-311
G	A	Homozygous	synonymous	GENE03321	hypothetical protein SERLA73DRAFT_171190	RL-LE-312
C	T	Homozygous	missense	GENE03324	-	
T	C	Homozygous	missense			RL-LE-314
T	A	Homozygous	missense	GENE03326	rho guanine nucleotide exchange factor scd1	
T	G	Homozygous	missense			
A	T	Homozygous	missense			
C	T	Homozygous	missense			RL-LE-316
C	T	Homozygous	missense	MNS3	Mannosyl-oligosaccharide 1,2-alpha-mannosidase MNS3	
A	C	Homozygous	missense			
T	C	Homozygous	missense			RL-LE-319
