# Supplementary figures and images for: Genome Sequencing of Lentinula edodes Revealed a Genomic Variant Block Associated with a Thermo-Tolerant Trait in Fruit Body Formation
Source: J Fungi (Basel). 2024 Sep 2;10(9):628. doi: 10.3390/jof10090628 (PMC11432811; doi:10.3390/jof10090628)

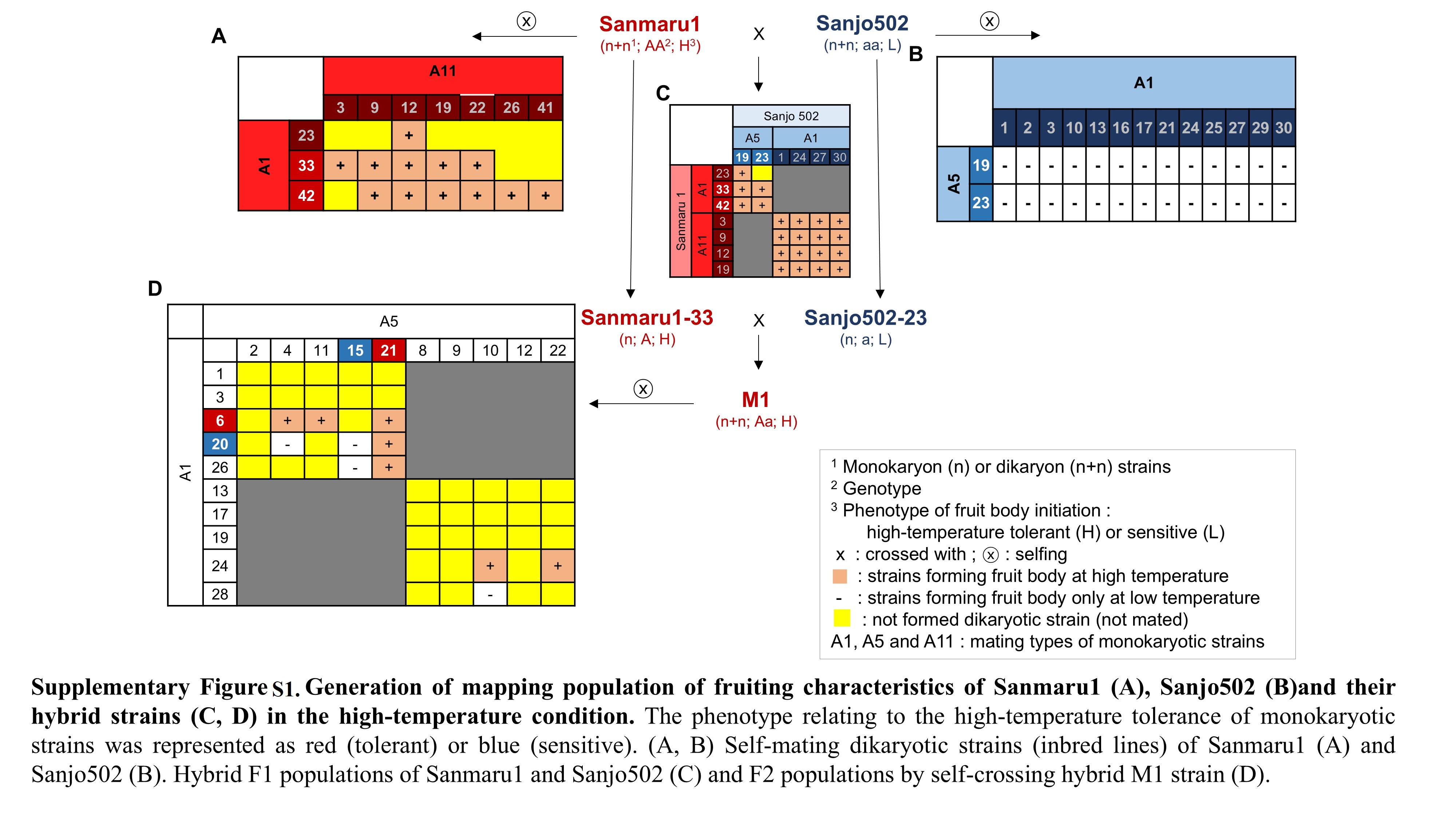

Supplement: Supplementary file 1 [file jof-10-00628-s001.zip › Figure S1.jpg]

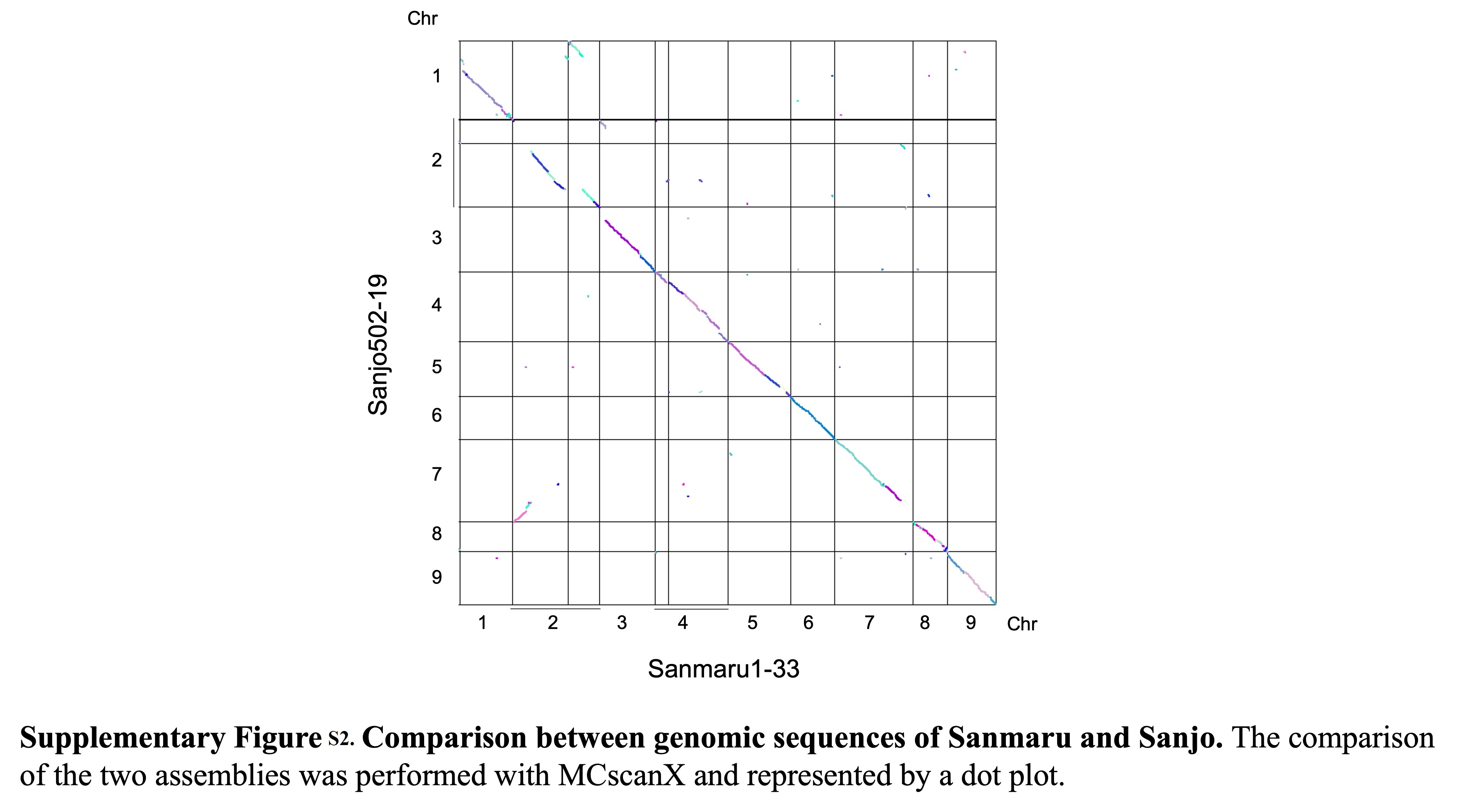

Supplement: Supplementary file 1 [file jof-10-00628-s001.zip › Figure S2.jpg]

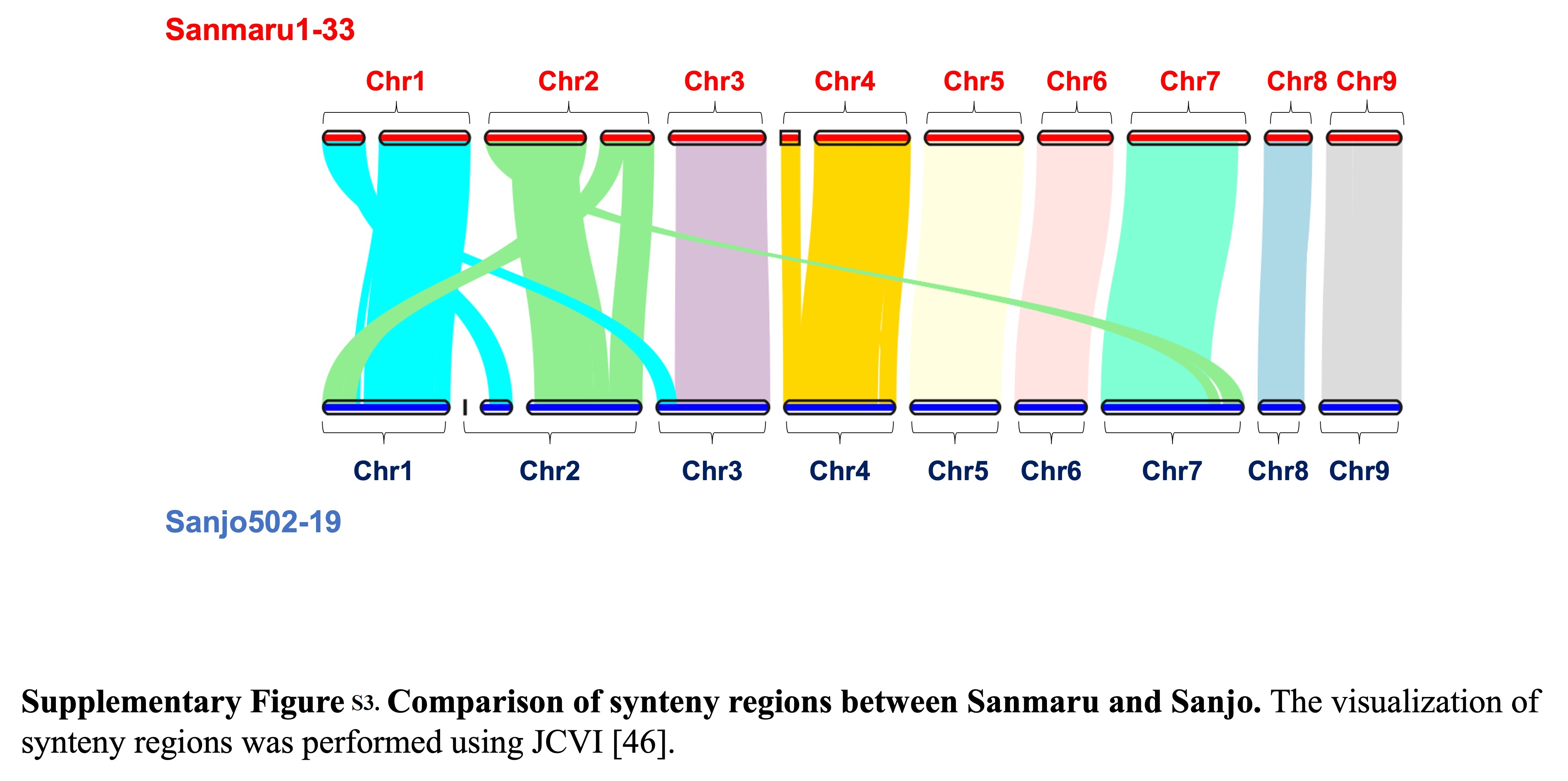

Supplement: Supplementary file 1 [file jof-10-00628-s001.zip › Figure S3.jpg]
